# Similarities and Differences Between Juvenile and Adult Spondyloarthropathies

**DOI:** 10.3389/fmed.2021.681621

**Published:** 2021-05-31

**Authors:** Corinne Fisher, Coziana Ciurtin, Maria Leandro, Debajit Sen, Lucy R. Wedderburn

**Affiliations:** ^1^Centre for Adolescent Rheumatology Versus Arthritis at University College London, University College London Hospital and Great Ormond Street Hospital, London, United Kingdom; ^2^Department of Adolescent Rheumatology, University College London Hospitals NHS Foundation Trust, London, United Kingdom; ^3^National Institute for Health Research University College London Hospitals Biomedical Research Centre, London, United Kingdom; ^4^Division of Medicine, Department of Rheumatology (Bloomsbury), University College London, London, United Kingdom; ^5^National Institute for Health Research Biomedical Research Centre at Great Ormond Street Hospital for Children, London, United Kingdom; ^6^Infection, Immunity & Inflammation Teaching and Research Department University College London Great Ormond Street Institute of Child Health, London, United Kingdom

**Keywords:** juvenile spondyloarthropathy, enthesitis related arthritis, adolescence, enthesitis, adult spondyloarthropathy

## Abstract

Spondyloarthritis (SpA) encompasses a broad spectrum of conditions occurring from childhood to middle age. Key features of SpA include axial and peripheral arthritis, enthesitis, extra-articular manifestations, and a strong association with HLA-B27. These features are common across the ages but there are important differences between juvenile and adult onset disease. Juvenile SpA predominantly affects the peripheral joints and the incidence of axial arthritis increases with age. Enthesitis is important in early disease. This review article highlights the similarities and differences between juvenile and adult SpA including classification, pathogenesis, clinical features, imaging, therapeutic strategies, and disease outcomes. In addition, the impact of the biological transition from childhood to adulthood is explored including the importance of musculoskeletal and immunological maturation. We discuss how the changes associated with adolescence may be important in explaining age-related differences in the clinical phenotype between juvenile and adult SpA and their implications for the treatment of juvenile SpA.

## Introduction

The spondyloarthropathies (SpAs) are a heterogeneous group of inflammatory arthropathies affecting both the peripheral and axial joints. In adults the most common form of SpA is axial SpA (axSpA) which includes ankylosing spondylitis (AS) and non-radiographic axSpA (nr-axSpA). Other forms of SpA include psoriatic arthritis, enteropathic arthritis, reactive arthritis, and undifferentiated SpA (1). Apart from axial and peripheral arthritis, common clinical features across all these subtypes can include enthesitis, dactylitis, and extra-articular manifestations such as acute anterior uveitis, inflammatory bowel disease (IBD), and psoriasis. There is a strong association between these conditions and the major histocompatibility complex (MHC) class 1 antigen human leucocyte antigen (HLA)-B27 although the mechanism for this association remains unclear (2). The prevalence of SpA varies widely by geographical area with the highest rates reported in North America and Europe which may correspond to the prevalence of HLA-B27 (3).

The onset of SpA occurs across a broad age range from childhood to adolescence and adulthood with a peak incidence in late adolescence and early adulthood (4). Although the common features remain the same, the clinical phenotype is different across the ages with peripheral arthritis and enthesitis predominant in juvenile SpA (JSpA) and axial manifestations more typical of adult onset disease. JSpA is classified differently from adult SpA as enthesitis related arthritis (ERA) which encompasses both axial and peripheral arthritis, and juvenile onset psoriatic arthritis, both of which are classified within the umbrella term juvenile idiopathic arthritis (JIA) (5).

The reasons for differences in clinical phenotype between adult onset disease and JSpA are not well-understood but may be due to the changes associated with musculoskeletal maturation and development of the immune system during childhood and adolescence. This review will discuss the similarities and differences between JSpA and adult onset SpA and highlight areas where better alignment between paediatric and adult disease would enhance research and clinical care.

## Classification Criteria

Adult SpA is classified by the Assessment of SpA International Society (ASAS) criteria which have been developed separating axial and peripheral SpA but encompassing the broad spectrum of disease including psoriatic arthritis, enteropathic arthritis, and reactive arthritis (Table 1) (6). There is a focus on the range of typical clinical features associated with SpA, including the association with HLA B27, enthesitis, and extra-articular manifestations. In addition, the inclusion of magnetic resonance imaging (MRI) assessment of the sacroiliac joints (SIJs) enables much earlier diagnosis of axial disease (including nr-axSpA) and therefore earlier treatment. This is in contrast to the older but still widely used modified New York criteria, which define AS by the grading of sacroiliitis using plain radiographs (7) and therefore capture more longstanding disease (8).

**Table 1:** Classification criteria for the diagnosis of adult SpA and JSpA.

**ASAS CRITERIA**	
**Axial SpA**			**Peripheral SpA**
For patients with ≥3 months back pain (with/without peripheral manifestations), aged <45 years			For patients with peripheral manifestations only
Sacroiliitis on imaging plus ≥1 SpA feature	OR	HLA-B27 plus ≥2 other SpA features	Arthritis or enthesitis or dactylitis plus
SpA features:			≥1 SpA feature:
• Inflammatory back pain • Arthritis • Enthesitis (heel) • Uveitis • Dactylitis • Psoriasis • Crohn's disease/Ulcerative Colitis • Good response to NSAIDs • Family history of SpA • HLA-B27 • Elevated CRP			• Uveitis • Psoriasis • Crohn's disease/ Ulcerative Colitis • Preceding infection • HLA-B27 • Sacroiliitis on imaging OR ≥2 other SpA features: • Arthritis • Enthesitis • Dactylitis • Inflammatory back pain ever • Family history of SpA
**ILAR JIA CRITERIA**		
**ERA**			**Juvenile onset psoriatic arthritis**
Arthritis AND enthesitis			Arthritis AND psoriasis
OR			OR
Arthritis OR Enthesitis plus ≥2 of:			Arthritis plus ≥2 of:	
• Current or past sacroiliac joint tenderness and/or inflammatory lumbosacral pain • HLA-B27 • Onset of arthritis in a male > 6 years old • Acute anterior uveitis • History of AS, ERA, sacroiliitis with inflammatory bowel disease, reactive arthritis or acute anterior uveitis in a first degree relative			• Dactylitis • Nail pits or onycholysis • Family history of psoriasis in a first degree relative
Exclusions:			Exclusions:
• Psoriasis or a history of psoriasis in the patient or a first degree relative • Presence of immunoglobulin M rheumatoid factor on at least two occasions 3 months apart • Presence of systemic JIA in the patient			• Arthritis in an HLA-B27 positive male beginning after their 6th birthday • AS, ERA, sacroiliitis with inflammatory bowel disease, reactive arthritis or acute anterior uveitis, or a history of one of these in a first degree relative • Presence of immunoglobulin M rheumatoid factor on at least two occasions 3 months apart • Presence of systemic JIA in the patient

*ASAS, Assessment of SpA International Society; ILAR, International League of Associations for Rheumatology; JIA, juvenile idiopathic arthritis*.

In contrast to adult SpA, JSpA is classified as part of the International League of Associations for Rheumatology (ILAR) classification criteria for JIA. JIA encompasses the broad spectrum of idiopathic inflammatory arthritis occurring before the age of 16 years, divided into seven mutually exclusive subtypes. Six subtypes are defined according to the pattern of joint involvement and extra-articular features during the first 6 months of disease. Those patients who do not meet the classification criteria for one of these six groups, or who meet the criteria for more than one subtype are classified in the seventh group as “undifferentiated JIA” (5). In contrast to adult SpA, patients with JSpA are not confined to one group but are divided between three subtypes of JIA: ERA, juvenile onset psoriatic arthritis and undifferentiated JIA (Table 1). The term ERA was developed in recognition of the distinct clinical presentation of childhood disease and replaced others including seronegative enthesopathy and arthropathy (SEA) (9), juvenile AS and JSpA.

There are well-recognised problems with the ILAR criteria (10–12). Compared to the ASAS criteria, the ILAR criteria lack the differentiation between axial and peripheral disease which is important for disease course and outcome. There is no inclusion of MRI assessment or inflammatory markers. The criterion of male sex in the ERA classification criteria increases the chance of diagnosis in males and leads to more females being classified as other JIA subtypes such as oligoarticular JIA. This may not be appropriate given recent evidence from studies in adults that the male predominance in SpA has significantly reduced in recent years (13). Patients with psoriatic arthritis and with a family history of psoriasis in a first degree relative are separated into the psoriatic arthritis category; despite these patients often having an overlapping disease phenotype with ERA (12). In addition, patients with enteropathic arthropathy and reactive arthritis are excluded from the psoriatic arthritis subtype (although are included in the ERA subtype) (5) even though they would all be considered as having SpA according to the adult ASAS criteria. A study of adult patients diagnosed with ERA in childhood found that 95% fulfilled the classification criteria for adult SpA (14).

In practice, the ILAR criteria result in a high proportion of patients with JSpA being classified in the undifferentiated JIA group and this, and the other reasons above, has led to a resurgence of the use of the term JSpA. The difficulties with the classification criteria and lack of alignment between paediatric and adult classification criteria has hampered the development of collaborative research studies in this field resulting in relatively few studies examining the clinical patterns, outcomes, and pathogenesis of JSpA.

## Pathogenesis

Research investigating the pathogenesis of JSpA is sparse compared to the extensive study of the pathogenesis of adult SpA. It is assumed that the pathogenesis is the same but studies including or comparing patients with adult SpA and JSpA are rare. Patient numbers in studies of JSpA are often small or are combined with other JIA subtypes and therefore it is difficult to draw conclusions from the current published data.

HLA-B27 is strongly associated with both adult SpA and JSpA and many subtypes exist. The most common allele is HLA-B27:05 which is associated with an increased risk of SpA across all races, ethnicities and ages (15). The HLA-B27:04 allele, which confers an increased risk in Eastern Asians was found to be the most prevalent subtype in a population of south Indian patients with ERA (16). Despite the strong association with SpA and in particular AS, HLA-B27 is only estimated to account for around 20% of the total heritability of AS (2, 17, 18) with other genetic risk alleles accounting for a further 8% (19). Many genetic variations related to pro-inflammatory pathways known to be associated with SpA have been found in patients with AS (20). After HLA-B27, genetic variations in endoplasmic reticulum aminopeptidase (ERAP) 1 are the most commonly recognised in adults (21) and have also been found in patients with ERA (22). Variations in the IL23 receptor (IL23R) gene are strongly associated with AS and other related conditions such as psoriasis and IBD (23) and have also been linked with juvenile onset psoriatic arthritis but not ERA (22). Differentially expressed genes in the mitogen-activated protein kinase (MAPK) pathway have been identified in both adult SpA and JSpA (24–26). Thus, similarities exist in the genetic associations between adult onset SpA and JSpA, but study of other genetic variants in the paediatric population is limited by low study participant numbers compared to the large numbers studied in adult SpA.

The interleukin (IL)23/IL17 pathway has been strongly implicated in the pathogenesis of SpA. IL23 plays a crucial role in enthesitis, the primary pathological feature of SpA (27). Increased production of IL23 is noted from macrophages of adult patients with AS compared to healthy volunteers (28) and IL23 is found in higher concentrations in the inflammatory lesions from facet joints of patients with AS compared to those with osteoarthritis (29). IL23 is less well-studied in ERA. One study demonstrated higher levels of IL23-producing intermediate monocytes in patients with ERA (30) but serum levels were no different between patients and healthy controls (31). Studies of serum IL23 levels in adult SpA have produced mixed results with some reporting higher concentrations and others no difference between patients and healthy controls (32–35). Recent studies suggest that IL23 may be critical in the early phases of disease pathogenesis (36) and therefore further investigation is important to understand its role in the pathogenesis of JSpA.

IL23 is crucial for the expansion and survival of Th17 cells (37) which are increased in the peripheral blood and synovial fluid of adult patients with SpA (38, 39). IL17 has also been found at higher concentrations in the serum and synovial fluid of adult patients with active SpA (40, 41). However, Th17 levels were not found in higher numbers in a small study of patients with ERA compared to controls (42) and levels of serum IL17 were no different between patients and healthy controls in another study by the same group (31). However, a subset of Th17 cells expressing the killer immunoglobulin receptor (KIR)3DL2, which interacts with aberrant forms of HLA-B27 and promotes Th17 cell survival, is increased in patients with ERA as well as adults with SpA compared to healthy controls (43). Levels of IL27, a regulatory cytokine in the IL23/17 pathway, are reduced in patients with ERA and this negatively correlates with Th17 cell concentration, suggesting dysregulation of the IL23/IL17 pathway is important in ERA (31). IL27 has been implicated in adult SpA through genetic studies (20).

Other pro-inflammatory cytokines such as tumour necrosis factor (TNF), IL6, IL1, and granulocyte-macrophage colony-stimulating factor (GMCSF) have also been implicated in the pathogenesis of SpA (44). The use of TNF blockade is highly effective in treating both JSpA and adult SpA therefore implying the importance of TNF in the pathogenesis of SpA across the ages. One study of synovial fluid TNF levels did not find any differences in levels between patients with ERA and another subtype of JIA (polyarticular JIA) or rheumatoid arthritis (45) but this is perhaps not surprising given the efficacy of TNF blockade across the inflammatory arthritides. In the same study, levels of IL6 were found to be higher in patients with ERA compared to those with polyarticular JIA. Increased serum levels of IL6 are found in patients with AS (46, 47) and IL6 is found in the inflamed SIJs of patients with AS (48). However, clinical trials of IL6 blockade in adults with SpA have failed to show clinical benefit (49) and therefore the significance of this is unclear. GMCSF has been implicated in the pathogenesis of inflammatory arthritis and in particular in the pathogenicity of Th17 cells (50) perhaps through a synergistic relationship with IL23 (51, 52). Higher numbers of GMCSF-producing Th17 cells are found in the peripheral blood of adult patients with SpA compared to patients with rheumatoid arthritis and healthy volunteers (53). GMCSF has also been implicated in oligoarticular and polyarticular course JIA but has not been studied specifically in JSpA (54).

Higher levels of interferon gamma (IFNγ) are found in synovial fluid from patients with ERA compared to those with polyarticular JIA (45). This is in contrast to adult SpA where lower levels of IFNγ are found in the synovium compared to those with rheumatoid arthritis and studies suggest dysregulation of IFNγ genes (55–57). Further study in both adult SpA and JSpA is important to understand the role of IFNγ in the pathogenesis of these conditions.

Chemokines have been studied in adult SpA as potential biomarkers for active disease and radiographic progression. Serum MMP3 levels have been shown to be higher in adult patients compared to healthy volunteers (58). In some studies, MMP3 levels reflect disease activity and may also predict structural damage (59). In contrast, for patients with ERA, MMP3 levels were found to be no different to healthy volunteers but did correlate with disease activity (60). MMP8 and 9 have been shown to be closely associated with disease activity in adult SpA in one study (61) but have not been studied in JSpA.

Another potential biomarker studied in both adult and JSpA is calprotectin or myeloid-related protein (MRP) 8/14. Serum levels are elevated in patients with SpA and may correlate with disease activity. High levels at baseline may predict radiographic progression and levels reduce on treatment with TNF inhibitors (62–65). Similarly in JSpA, plasma levels have been shown to be higher in patients compared to healthy volunteers; high levels correlate with active disease and reduce in those who respond to treatment (66). MRP8/14 is helpful in predicting response to treatment in patients with JIA (67, 68). In addition, faecal calprotectin is an established biomarker of disease activity in inflammatory bowel disease (69) and is elevated in adult patients with SpA (70). One small study of patients with ERA demonstrated higher levels compared to disease control patients with connective tissue diseases but further study is needed (71).

Evidence for the involvement of gut flora in the pathogenesis of SpA dates back many years with gram negative bacteria implicated in particular in both adult SpA (72–75) and JSpA (76). Microbes found in the gut shape host immune response from an early age (77) and several studies have shown differences in the gut microbiome between patients with SpA and healthy controls. A study by Costello et al. found differences in certain families of bacteria from terminal ileal biopsies from patients with AS (increases in Lachnospiraceae, Ruminococcaceae, Rikenellaceae, Porphyromonadaceae and Bacteroidaceae and decreases Veillonellaceae and Prevotellaceae) compared to healthy volunteers (78). Another study demonstrated an increase in Ruminococcus gnavus in faecal DNA from adult patients with SpA compared to those with rheumatoid arthritis and healthy volunteers (79). A large Norwegian cohort study revealed increased Proteobacteria, Enterobacteriaceae, Bacilli, Streptococcus species, and Actinobacteria, but lower abundance of Bacteroides and Lachnospiraceae in patients with AS compared to healthy volunteers. A link with raised faecal calprotectin was observed in patients with a lower abundance of certain bacterial species with anti-inflammatory properties (80). The microbiome has also been studied in JSpA with one study of Indian patients showing similar findings to studies in adult SpA with increases in Bacteroidaceae and Enterobacteriaceae families and a reduction in the Prevotellaceae family in patients compared to healthy controls. A study of both adult patients with SpA and patients with ERA found that a strain of the anti-inflammatory bacterial family Faecalibacterium prausnitzii was reduced in both patients with ERA and SpA. However, a higher abundance of Bacteroides was noted in patients with ERA compared to SpA (81) and this has also been found in other JIA subtypes (82). The presence of HLA-B27 appears to influence the gut microbiome (83). In addition, changes in the gut microbiome may result in upregulation of Toll Like receptors (TLRs) causing an inflammatory cascade and the production of pro-inflammatory cytokines (84). The expression of TLRs 2, 4, and 5 is upregulated in the synovium and peripheral blood mononuclear cells (PBMCs) of patients with SpA (85–87). Upregulation of TLRs 2 and 4 has also been found in patients with ERA (88).

Therefore, despite the similarities between the pathogenesis of adult SpA and JSpA, some differences exist. These may be explained by the lack of studies directly comparing adult SpA and JSpA or are perhaps due to lower patient numbers in studies of JSpA. However, a study comparing synovial biopsies in patients with JSpA, including both patients with ERA and juvenile onset psoriatic arthritis, to biopsies from patients with adult SpA found similarities but also marked differences. In particular, in JSpA there was a stronger lining layer hyperplasia and the number of infiltrating CD163+ macrophages were lower which meant JSpA failed to classify in the SpA group by class prediction analysis. Instead, there was partial overlap with other JIA subtypes suggesting that age may strongly influence the pathogenenic features of SpA (89). Factors such as changes in the immune system and gut microbiome with age, which have been demonstrated in detailed studies of healthy subjects and in smaller cohorts of patients (90, 91) may give rise to some of the differences described above and distinct clinical phenotype of JSpA compared to adult SpA. Some specific immune age-related changes have been demonstrated to occur at the time of puberty in both boys and girls such as IFN production in response to TLR stimulation (92). However, how these age-related immune function changes relate to the differences between JSpA compared to adult SpA, remains an area for future investigation.

## Clinical Features

Multiple studies across different countries clearly demonstrate the predominance of peripheral arthritis and enthesitis in JSpA compared to adult SpA where axial arthritis is the most common clinical feature at onset and throughout disease course (93–99). Shoulder and hip joint involvement are also more common in JSpA (100) and the most frequently involved joints in JSpA include the knee (40–50%), ankle (25–40%), and hip (30–40%) (101, 102). As in adult SpA, enthesitis commonly affects the lower limb in JSpA and, in particular, the inferior pole of the patella, plantar fascial insertion into the calcaneum, Achilles tendon and the tibial tuberosity (103–105). In one study, enthesitis was shown to be present in 83% of patients with ERA on ultrasound but clinical examination yielded a much lower detection rate (106). Enthesitis is a key feature of early disease in SpA and the lack of sensitivity of clinical examination may contribute to diagnostic delay. This has been identified to be longer in patients with JSpA compared to adult SpA in several studies (96, 97, 107) although not consistently (95).

In general, comparisons between JSpA and adult SpA have not identified significant differences in the male to female ratio, HLA-B27 status, family history of HLA-B27-related disease and extra-articular manifestations such as psoriasis and inflammatory bowel disease. An increase in uveitis has been reported in JSpA compared to adult SpA in a small number of studies (97, 98) but this was not confirmed in a recent meta-analysis (108).

## Assessment of Disease Activity

Disease activity is measured differently in adult SpA and JSpA. The AS disease activity score (ASDAS) is now the most widely used assessment of disease activity in adults (109) and includes patient-reported measures of back pain, morning stiffness, peripheral joint pain and swelling, global assessment, and a measure of inflammation (either ESR or CRP) (Table 2). It has validated classifications for inactive, low, high, or very high disease activity (110) but has not been validated in JSpA. Other disease activity measures in use in adults include the Bath Ankylosing Spondylitis Disease Activity Index (BASDAI) (111) and the Bath Ankylosing Spondylitis Functional Index (BASFI) (112). These have been validated in children and young people but focus on spinal disease and underestimate enthesitis, therefore may not accurately reflect disease activity (113, 114).

**Table 2:** Comparison of adult and paediatric disease activity scores for SpA.

**AS disease activity score (ASDAS)**	**Juvenile SpA disease activity (JSpADA) index**
- Back pain - Peripheral pain/swelling - Morning stiffness - Patient global assessment - CRP/ESR	- Active joint count - Active enthesitis count - Patient pain score - CRP/ESR - Morning stiffness - Clinical sacroiliitis - Uveitis - Back mobility (modified Schober's test)

*CRP, C reactive protein; ESR, erythrocyte sedimentation rate*.

Until recently, disease activity in JSpA was measured using the same scores used for all JIA subtypes. The two scores in common use are the juvenile arthritis disease activity score (JADAS) which measures four domains including physician global assessment, patient global assessment, active joint count, and an inflammatory marker (either CRP or ESR) (115) and the core outcome variables which include a physician global assessment, patient/ parent global assessment, functional status, inflammatory marker (ESR), number of joints with active arthritis, and number of joints with restricted range of movement (116). However, neither of these measures assesses enthesitis or axial arthritis and therefore disease activity in patients with JSpA is likely to be underestimated. The need for a dedicated disease activity score for JSpA has been met with the development of the juvenile SpA disease activity (JSpADA) index. This assesses 8 domains including active joint count, enthesitis count, patient pain assessment, inflammation (either CRP or ESR), morning stiffness, clinical evaluation of sacroiliitis, uveitis, and back mobility measured by the modified Schober's test (Table 2). Necessarily, for the paediatric population, it contains more objective measures and fewer patient reported measures compared to the ASDAS. It has undergone preliminary validation in a patient population with a mean age of 12 years and appears to be superior to other JIA disease activity measures in assessing disease activity in ERA (117). It has been subsequently validated in other populations with an older mean age (14.3 years) (113) and has started to gain wider use.

Further measures of disease severity in adult SpA include the Bath Ankylosing Spondylitis Metrology Index (BASMI) which measures spinal movement and the modified Stoke AS Severity Score (mSASSS) which evaluates disease severity on plain radiographs. However, neither of these measures is useful in JSpA as they focus predominantly on axial disease and are not sensitive for early SpA or peripheral arthritis (118, 119). Radiographic changes seen on the mSASSS are a reflection of late disease and plain radiographs of the SIJ in children and young people are often misleading with frequent false negative and false positive results (120).

Imaging, in particular MRI, is useful to assess disease activity and severity in axial disease, peripheral arthritis, and enthesitis in both adult SpA and JSpA. MRI features of axial SpA include both inflammatory lesions such as bone marrow oedema and osteitis and structural lesions such as erosions, sclerosis, and bony ankylosis. These features alone lack specificity and must be interpreted together with the clinical picture (121–123). Caution is needed in the interpretation of MRI of the SIJs in children and young people as significant variability is found (124). Features of sacroiliitis are comparable to those found in adults (125) although diagnostic criteria used in adults may have a lower sensitivity in children and young people (126). More significantly, MRI images of normal marrow and cartilage development can be mistaken for bilateral sacroiliitis. In particular, bilateral symmetrical metaphyseal high signal and cortical irregularities along the ilial margin of the SIJ which occur with normal maturation may appear similar to sacroiliitis leading to misdiagnosis of JSpA (127, 128). This was illustrated in a multicentre study in which significant variation in the interpretation of inflammatory and structural lesions on MRI of the SIJ in patients with JSpA was seen giving rise to frequent false positive results (129). Another single centre study showed low to moderate inter-reporter reliability when interpreting SIJ MRI in children and young people (130). Clinical examination, back pain and buttock pain all have a low sensitivity for identifying sacroiliitis in children and young people (131) and therefore novel MRI techniques which help to distinguish true sacroiliitis from the changes associated with normal maturation in children and young people are important (132).

Other imaging modalities such as ultrasound are helpful to assess enthesitis which may be difficult to detect clinically (121, 133). However, again, caution is needed in the interpretation of ultrasound findings in children and young people. There is significant variability in entheseal thickness with growth and development, making it difficult to define a “normal range.” One study in healthy children revealed a correlation between entheseal thickness and age (134). However, another study found a correlation with weight. This study also noted that boys tended to have thicker entheses than girls but significant variability was found even between left and right sides in the same subject (135).

## Treatment

Multidisciplinary management is important in the treatment of both adult SpA and JSpA. There is good evidence for physiotherapy intervention in adult SpA but no studies in JSpA. However, the impact of diagnosis and symptoms (as well as delayed diagnosis) is often significant on psychosocial well-being as well as education and physical functioning in children and young people (136, 137).

Non-steroidal anti-inflammatory drugs (NSAIDs) are the first line pharmacological therapy in adult SpA and improve symptom in both axial and peripheral arthritis (138). There is some evidence to suggest prolonged, continuous use of NSAIDs may slow radiographic progression in adult SpA (139, 140) but this was not confirmed in a prospective study (141). NSAIDs are also frequently used as first line therapy in JSpA (105, 142) but there is no evidence for continuous or prolonged treatment or of the effect on disease progression. Whilst rarely used in adult SpA, local, or systemic corticosteroids are sometimes used as a holding measure before treatment with either DMARDs or TNF inhibitors is effective in JSpA, although they may not be as effective as in other JIA subtypes.

Conventional disease modifying anti-rheumatic drugs (DMARDs) such as methotrexate and sulfasalazine are only used if there is peripheral arthritis in adult SpA (143, 144) as they are ineffective in axial disease (145, 146). However, DMARDs are commonly used in JSpA because of the high prevalence of peripheral arthritis and enthesitis. Although methotrexate is the most commonly used DMARD for JIA (including ERA and psoriatic arthritis), there is no specific evidence for the use of methotrexate in JSpA as patients with ERA and psoriatic arthritis were excluded from a large trial of methotrexate in patients with JIA (147). Sulfasalazine was found to be effective in a randomised double blind placebo controlled trial in 33 patients with JSpA after 26 weeks treatment (148).

When the treatments above do not establish adequate disease control, TNF inhibitors are highly effective treatments for adult SpA and JSpA, especially for axial arthritis where conventional DMARDs are not effective. In adults, numerous randomised controlled trials (RCTs) have provided good evidence for the efficacy of etanercept (149), adalimumab (150), infliximab (151), golimumab (152), and certolizumab (153) with reported response rates of around 60%. Until recent years, the efficacy of TNF inhibitors in JSpA was established through open label studies, retrospective analyses and reports from registries. Treatment regimens for JSpA were often the same as those of other JIA subtypes without stratification between axial and peripheral disease. However, two RCTs of treatment in JSpA have been published demonstrating the efficacy of etanercept (154) and adalimumab (155). Adalimumab is effective for the treatment of axial disease in JSpA (156) and for treating extra-articular manifestations including uveitis (157), inflammatory bowel disease (158), and psoriasis (159). Of the other TNF inhibitors, only Infliximab has been studied specifically in JSpA in one randomised trial (160). There does not appear to be any difference in the efficacy of the different TNF inhibitors in the treatment of adult SpA and JSpA but there is evidence that the monoclonal antibodies (adalimumab, infliximab, golimumab, certolizumab) are superior to etanercept in the treatment of extra-articular manifestations and therefore may be used in preference when these are present (161, 162).

In both adult SpA and JSpA, there is evidence that early treatment with TNF inhibitors may be more beneficial with the possibility of partial remission demonstrated in adults with axSpA (163, 164) and a greater improvement in disease activity and lower reported pain scores in patients with JSpA (165). Following the initiation of TNF inhibitors, sustained remission without treatment is rare in both adult SpA and JSpA, even in those treated early, with most patients relapsing within a year of treatment cessation (166–169). However, tapering treatment by increasing the interval between doses or reducing the dose may be possible (170–172).

Despite the clear benefit of treatment with TNF inhibition, the effect on radiographic progression has been debated in adults with axSpA. Several studies have demonstrated continued new bone formation despite treatment (173–175). However, more recent studies do suggest that TNF inhibitors slow radiographic progression, especially if used early in disease, suggesting a potential early window of opportunity for treatment (176–179). In JSpA, one small study demonstrated progression of sacroiliitis despite treatment with TNF inhibitors (180) and SIJ bony ankylosis occurs with rates correlating with the length of time taken to start treatment with TNF inhibition (181).

Given the importance of the IL23/IL17 pathway in the pathogenesis of SpA, biologics inhibiting this pathway are of particular interest for the treatment of patients with SpA. Ustekinumab, an inhibitor of IL23, was initially found to be effective in an open-label study of patients with active AS (182). However, no benefit was found in two subsequent RCTs, and the studies were terminated (183). Ustekinumab is an effective treatment for psoriatic arthritis (184) including axial arthritis in this group, and is also an effective treatment of enthesitis in patients with psoriatic arthritis (185). In JSpA, a case series of five patients treated with Ustekinumab demonstrated an improvement in disease activity in all but one patient (186) but no RCTs have been carried out. Other inhibitors of IL23, including guzelkumab, are effective treatments for psoriasis and psoriatic arthritis and demonstrate significant improvement in enthesitis (187). However, no benefit has been found in the treatment of axSpA and there have been no studies in JSpA (188). Given that enthesitis is an early feature of SpA and that there is a high prevalence in JSpA, investigating the effect of IL23 inhibition in patients with JSpA should be considered.

Treatments inhibiting IL17 have proved more effective than those inhibiting IL23 in adult SpA and for axSpA in particular, with response rates similar to those of TNF inhibitors (189). Secukinumab is now an established treatment for adults with SpA (including psoriatic arthritis) (190) and a clinical trial in JSpA has recently been completed (ClinicalTrials.gov: NCT03031782). Other agents blocking IL17 such as ixekizumab and brodalumab have proved effective for axSpA and psoriatic arthritis (191–193) and a clinical trial of ixekizumab is planned in JSpA (ClinicalTrials.gov: NCT04527380). It has been suggested that IL17 inhibitors may slow radiographic progression more effectively than TNF inhibitors (194) but this needs further investigation with “head to head” studies.

JAK inhibitors are another emerging treatment for SpA and Tofacitinib has shown similar efficacy to TNF inhibitors in adult SpA, including axSpA and psoriatic arthritis (195, 196). Clinical trials of JAK inhibitors are underway in patients with JIA, including patients with ERA and psoriatic arthritis subtypes (ClinicalTrials.gov: NCT02592434, ClinicalTrials.gov: NCT03773978). GMCSF inhibition is also under investigation in axSpA but not yet in JSpA (ClinicalTrials.gov: NCT03622658). Other biologic agents including IL6 and IL1 receptor inhibitors (197, 198), abatacept (199), and rituximab (200) have proved ineffective for the treatment of adult SpA and have not been studied in JSpA.

## Outcomes

Factors associated with a worse prognosis in adult patients with SpA have historically included the presence of hip arthritis and three or more of persistently raised ESR, unresponsiveness to NSAIDs, limitation of the lumbar spine movement, dactylitis, oligoarthritis, or onset younger than 16 years (201). AxSpa, the most studied form of SpA, is noted to be more common in men and is associated with higher levels of inflammation, more structural changes on MRI, higher levels of peripheral arthritis and enthesitis, and more uveitis compared to those with nr-axSpA (202). HLA-B27 status and severe inflammation on SIJ MRI in early disease are predictors of more severe radiographic changes after 8 years follow up (203). Obesity is associated with a worse outcome in axSpA (204). Differences in outcomes between males and females are reported (13).

There are relatively few outcome studies in JSpA and most compare JSpA to other JIA subtypes. These studies demonstrate that JSpA, in particular ERA, has low rates of remission and a worse prognosis compared to most other subtypes (205, 206). Despite treatment with TNF inhibitors and good initial response rates, this has not changed in recent years with ongoing disease activity seen in around 60% (142, 207). Higher pain scores and worse levels of physical function are also seen in patients with ERA compared to other JIA subtypes (208–210). Factors influencing prognosis in JSpA are similar to those in adult SpA. HLA-B27 in patients with JIA is associated with an increased chance of developing inflammatory lower back pain and, in males, enthesitis, and a lower chance of clinical remission after 8 years (211, 212). Other factors associated with a worse prognosis include male sex, ankle and hip arthritis and persistently raised inflammatory markers (206). Two studies comparing JSpA and adult SpA reported a greater requirement for hip arthroplasty in patients with JSpA (213, 214).

## The Effect of Adolescence: Sex Hormones and Mechanical Stress

The peak age of onset of JSpA differs from most other JIA subtypes and corresponds to the onset of adolescence. Some HLA-alleles have been associated with early childhood arthritis. However, HLA-B27 appears to confer protection from the development early onset disease and has been associated with later onset arthritis in children which may explain the peak age of onset in early adolescence (215).

Increased oestrogen and progesterone production in females and increased testosterone production in males occur at the time of puberty. The effect of sex hormones on the immune system is well-documented and key changes in adolescence include higher levels of monocytes in males compared to females (216) and higher levels of T cells in females (217). The gut microbiome is also unique in adolescents and increased diversity of microbial species has been noted compared to adults. Sex hormones are also associated with differences in gut microbiota between males and females (218). Sexual dimorphism in adult SpA is well-recognised with higher levels of circulating Th17 cells in males with AS, in addition to higher serum levels of IL17, TNF, and IL18 (219, 220). Differences are also noted in clinical outcomes and response to treatment. In SpA, male sex is associated with more extensive bone marrow oedema of the SIJs (221) and a worse radiographic outcome (222–224). However, female sex is associated with higher disease activity and pain scores (222, 223, 225–227), less severe radiographic progression (222, 228–232) and a lower response rate to treatment with TNF inhibitors (233–235). These aspects have not been studied in JSpA but given the typical age of onset around the time of puberty, and the effect of sex hormones on the immune system and gut microbiome, it seems likely these would influence disease pathogenesis.

Adolescence is also a time of significant skeletal development and therefore increased mechanical strain on the musculoskeletal system. SpA tends to affect sites under the most mechanical strain such as the lower limb and the spine. Extra-articular manifestations of SpA are also found at sites of mechanical stress such as the aortic root and anterior uveal tract (236). In adult SpA, studies have shown more severe disease in those with manual jobs and with certain types of exercise (237, 238). One study of patients with JSpA showed nearly a quarter took part in intense physical training (95).

Enthesitis, the primary lesion in early SpA, occurs in susceptible individuals following repetitive microtrauma (239). Mechanical stress has been shown to exacerbate enthesitis in animal models and results in the release of pro-inflammatory cytokines at the enthesis (240). In heathy individuals, the resultant recruitment of innate immune cells leads to healing. However, in SpA, perpetuation of the inflammatory response occurs, perhaps due to IL23/IL17 pathway activation, HLA-B27 or gut dysbiosis, resulting in aberrant tissue healing and eventual new bone formation (241). Mechanical stress is heightened during periods of growth and development which may explain the prevalence of enthesitis and peripheral arthritis in children and young people with JSpA. Anatomical studies of the developing skeleton during puberty show that the most important factor in sacral development is the effect of mechanical force which occurs through body weight, load through the femur and strain on the pubic symphysis. Similarly, normal SIJ development is dependent torsion between the ilia and the sacrum (128, 242). Changes in SIJ orientation are seen with age and significant differences are found between adults and children and adolescents (243). In addition, distinct pelvic morphology develops in males and females after puberty with larger SIJ surface area in males thought to be related to higher biomechanical loading and larger ligamentous attachments (244). These changes in biomechanical loading and mechanical stress which occur during skeletal development may, in susceptible individuals, result in inflammation at the entheses and later at the SIJs.

## Conclusion

There are many similarities between adult SpA and JSpA suggesting that they are a spectrum of the same disease. Key differences in classification criteria, prevalence of clinical features, disease activity, and pathogenesis are evident which are influenced by age, pubertal development, changes in the immune response, and skeletal maturation. Further research is needed in to these factors which may influence treatment stratification for patients with JSpA. Better alignment between paediatric and adult classification and assessment criteria as well as studies encompassing the whole age spectrum of SpA will further our understanding and improve the treatment of this important disease.

## Author Contributions

This article was drafted by CF and reviewed and revised by CF, CC, ML, DS, and LW. All authors agree to be accountable for the content of the work.

## Conflict of Interest

The authors declare that the research was conducted in the absence of any commercial or financial relationships that could be construed as a potential conflict of interest.
